# Asylums of the World. III.—Administration and Nursing


**Published:** 1892-07-16

**Authors:** 


### The Nursing.

### INSTITUTION LITERATURE.

### ASYLUMS OF THE WORLD.*

#### III.—Administration and Nursing.

A spirited article on lunacy in Ireland, in the book before us, exposes the many defects and inconsistencies of lunacy administration in that country, and gives us rather a sombre picture of the condition of the Irish asylums and their management. We are told that "a spirit of economy prevails which in other countries would not be considered consistent with due provision for the insane." We read: "It is probable that no workhouse in England presents so gaunt and cheerless an appearance as may yet be found in many Irish asylums." Nor do the private asylums appear in a better light. One of them especially seems to have been most unhappy in its management, and extremely retrograde in the treatment of its inmates. "The institution (St. Patrick's) was one of the latest strongholds of the worst forms of restraint. The present Superintendent, on taking charge of the institution some six years ago, appears to have done what Pinel did in France and William Tuke in England a hundred years before." Several anomalies in the present system of asylum administration call urgently for redress. Chief of these is the appointment and function of visiting physicians, one of whose duties appears to be to sign certificates of discharge, along with the medical superintendent, in the case of every patient who leaves the asylum. It thus comes about that in Ireland two medical certificates are required for the discharge of a patient from an asylum, while one certificate is sufficient to admit him. It is, therefore, twice as difficult to get a patient out of an asylum as to get him into one. The assistants to the resident medical superintendents are appointed by the Government. As they are appointed not upon merit so much as by influence, after vigorous canvassing, we are told that "it is not to be wondered at that some of the most sagacious superintendents have not pressed for the assistance which might otherwise be thought necessary." It thus comes about that only ten of the twenty-two district asylums possess assistant medical officers. It is with satisfaction, therefore, that those interested in lunacy administration throughout Great Britain and Ireland will peruse the admirable recommendations of Sir Arthur Mitchell's Committee for the amelioration and reconstruction of lunacy legislation in Ireland. With some exceptions, a few of which we have endeavoured to embody in this short notice, the asylums of Great Britain and Ireland, so far as their management and administration are concerned, may be considered good as a whole. It is impossible to lay down any hard and fast rule by which the medical staff which forms the guiding, controlling influence, as well as the source of the status and tone of the institution, should perform its function. So much will depend upon idiosyncrasy, upon opinion, and upon the interpretation which each medical superintendent attaches to the duties of his office, that allowance must be made for extreme divergence in the medical administration of the various asylums throughout the country. If the superintendent considers himself the governor of a colony of incurable lunatics, he will interpret his function in one way; if he regards himself as the physician of a hospital for the cure of insanity he will interpret it in quite another way. In the pages before us we have the following very correct exposition of the question: "As the old prison idea of an asylum is vanishing rapidly from the minds of the people, and the keeper is giving way to the attendant and nurse, so we hope soon to see all our county and other asylums bearing the name of hospitals for the insane, but by

* "Hospitals and Asylums of the World." By Henry C. Burdett Vols. I. and II.—Asylums and Asylum Construction. (London: J. and A. Churchill.)

whatever name these institutions are henceforth to be known, it cannot be too strongly insisted upon, or too constantly borne in mind, that they must ever and always be looked upon as places where a serious brain disease is to be treated, and that it is the individual treatment as opposed to the mass treatment, that marks the best asylum in our own day. It is further to be hoped that individual treatment will still more distinctly mark those of a succeeding age." With the general tone and aspiration of a paragraph such as this, we are in entire sympathy; but we fail to see how, under the present monstrous system of accumulating together from 1,000 to 2,000 lunatics under the care of one superintendent, with two or three medical assistants, such a thing as individual treatment can possibly be expected. We are confidently of opinion that the most certain method of insuring individual treatment and hospital treatment is the establishment of separate hospital blocks in connection with every asylum, and the limitation of the insane population of each asylum within such a reasonable number as can receive the individual attention of one medical head. In order to establish this or any other system of medical or individual treatment, we are of opinion that some means should be adopted for placing in separate industrial colonies the congenitally feeble-minded and the hopelessly chronic insane. Abundant work for the medical energies of asylum officers, and an outlet for the nursing instincts of the staff, would remain attached to the present asylums in the treatment of the acutely insane—of the noisy, non-industrious chronic patients, and of the infirm and physically incapacitated patients.

With the aid of a sufficient staff of subordinate officers the administrative department of an asylum should, under ordinary circumstances, be capable of working smoothly in the groove of routine, and it should be sufficient for the medical superintendent to remain in constant touch with the working of the institution without any inordinate tax upon his time or his strength. The burden of his duty lies in the direction of the medical treatment of his patients, and with this end in view the whole tone and character of each department in the asylum should be tinged, and all the energies of its officials devoted to this one chief purpose. He should be conversant with the literature of his specialty; he should impart to his medical assistants a devotion for their special work, and instil into his nursing staff an enthusiasm for the art of healing for its own sake. The idea that should strike a visitor in entering such an institution should be, "This is not a prison for the confinement of lunatics, but a medical establishment for the treatment of insanity."

The training of attendants and nurses for the special care of the insane is a feature of the latest development of asylum administration. No advance in the treatment of the insane can now be made without the intelligent, conscientious, we might add enthusiastic co-operation of the subordinate staff.

"What obstructs, mars, subverts therapeutical and moral treatment and the plainest dictates of humanity? They (physicians and superintendents) would answer unanimously that those obstacles consist in the inefficiency of their instruments" (p. 631). Now that the pay is improved, the comforts of attendants in most asylums secured, the National Pension Fund available for their protection against illness and old age, the status and position of our attendants ought to rise. To procure suitable and good attendants for asylums is the desideratum of every asylum superintendent. The lack of them is the great drawback of the modern medical asylum system for the treatment of the insane.

Much has been written and spoken upon the subject; much remains still to be said and done. The ideal attendant has still to be found, and although the type is improving slowly, we fear that the improvement is not an actual but a relative one—relative we mean to the educational and social improvement in the general mass of the people. Nearly forty years ago hospital nursing was in as bad or worse a condition than asylum nursing now is. Florence Nightingale became the apostle and moving spirit of a new system, which has now left little to be desired in this respect, throughout the length and breadth of our land.

The movement stopped short at asylums with such a sharp line of demarcation that to this day not a ray of its enthusiastic light has directly penetrated their gloom, nor has there arisen with asylums themselves any corresponding movement nor any apostle of a new order of things.

We ask why this unhappy condition exists, and a host of replies greet us from every side. "The work is repulsive," so is nursing the sick to the carnal mind; "the hours are long," nurses of the sick have no hours; "the pay is inadequate." Is the hospital nurse, or the medical practitioner, or the missionary to the heathen, or scientific labourer adequately paid? By no means.

If we inquire whether attendants upon the insane are individually or collectively equal in moral excellence, in individual worth in industry and high appreciation of duty to people of the same class as themselves who enter into trades, into business of various kinds, even into domestic service, we find that they are not. The following quotations from the late Dr. W. A. F. Browne, one of the Commissioners in Lunacy for Scotland, and from the writings of Dr. Clouston, the well-known Scottish authority, both of whose opinions are reproduced in the work before us, speak for themselves.

Dr. Browne says (p. 634) "they are drawn from the large class who covet the luxuries of life without its labours or what is luxury to them, good food and raiment and an easy loitering employment in which the unscrupulous may make long intervals of rest alternate with brief periods of activity and worry, who economise physical exertion, and rarely attempt to acquire the moral influence which is won by kindness and judgment. They are the lazy bodies, the rejected, the outcasts of other trades."

Dr. Clouston says (p. 640) that the class of persons who offered themselves for the post of asylum attendant "were usually the sort of men who are too idle to work hard and the sort of women who go as servants to hotels and lodging-houses."

Since Drs. Browne and Clouston wrote, a change for the better has, as we have already indicated, taken place. But a satisfactory solution of this difficult question seems as distant as ever.

Recently the Medico-psychological Association has done good work in the right direction by establishing a certificate for attendants and nurses, which is only obtainable after long service, special training and examination. As to the utility of such a step there need be no question, but whether it will prove a cure for the existing evil is open to doubt.

The desiderata for a good asylum attendant are suitability of temper, education, industry, and a certain amount of moral force. Why do those most frequently deficient in those very qualities seek asylum service, while those possessed of them prefer harder work, and, it may be, equally inadequate remuneration?

The answer must be that the tendency of asylum work is not so morally elevating nor generally regarded as so respectable as other forms of labour and employment.

What is required is *esprit de corps*, and that can only be borne with a new evangel and under the influence of an apostle. Once establish a system, once let it become honourable and morally elevating, and above the power of the critical public to despise, and the evils existing vanish, and Dr. Clouston's ideal, and the ideal of every good medical superintendent is realized. It will not be realized in a day, but will spread and overcome prejudice and error as every other high and true ideal has done. Then, and not till then, will the prison character of asylums vanish and the gaoler give place to the nurse.

Will no asylum committee or asylum superintendent with exceptional opportunities take the lead and redeem us from the tyranny and the oppressive shadow of a system chronologically and morally defunct?

